# Association of brachial–ankle pulse wave velocity and carotid plaque in Chinese hypertensive adults: effect modification by age

**DOI:** 10.1038/s41440-020-0432-2

**Published:** 2020-04-17

**Authors:** Zhihao Liu, Ying Yang, Yan Zhang, Liling Xie, Qinqin Li, Yun Song, Lishun Liu, Chengzhang Liu, Benjamin Xu, Binyan Wang, Tieci Yi, Fangfang Fan, Wei Ma, Haoyu Weng, Nan Zhang, Xiping Xu, Xiaobin Wang, Xianhui Qin, Yong Huo, Jianping Li

**Affiliations:** 1 Department of Cardiology, Peking University First Hospital, 100034 Beijing, China; 2 National Clinical Research Study Center for Kidney Disease, State Key Laboratory for Organ Failure Research, Renal Division, Nanfang Hospital, Southern Medical University, Guangzhou, 510515 China; 3 Beijing Advanced Innovation Center for Food Nutrition and Human Health, College of Food Science and Nutritional Engineering, China Agricultural University, 100083 Beijing, China; 4 Institute of Biomedicine, Anhui Medical University, Hefei, 230032 China; 5 Shenzhen Evergreen Medical Institute, Shenzhen, 518057 China; 6 Trinity College of Arts and Sciences, Duke University, Durham, NC 27708 USA; 7 Department of Population, Family and Reproductive Health, Johns Hopkins University Bloomberg School of Public Health, 615N. Wolfe Street, E4132, Baltimore, MD 21205-2179 USA; 8 Key Laboratory of Molecular Cardiovascular Sciences of Ministry of Education, Health Science Center, Peking University, 100083 Beijing, China

**Keywords:** Brachial–ankle pulse wave velocity, Carotid plaque presence, Carotid plaque number, Hypertension, Age

## Abstract

We aimed to investigate the association of brachial–ankle pulse wave velocity (baPWV) with carotid plaque presence and carotid plaque number in a Chinese hypertensive population. A total of 13,554 hypertensive subjects from the China Stroke Primary Prevention Trial (CSPPT) were recruited. Arterial stiffness and carotid plaque were evaluated by baPWV and B-mode ultrasonography, respectively. Multivariate logistic regression analysis was used to determine the correlation of baPWV and carotid plaque presence. Multinomial logistic regression analysis was used to determine the correlation of baPWV and carotid plaque number. Further interactions between baPWV and carotid plaque presence were examined using subgroup analysis. Continuous baPWV was positively correlated with carotid plaque presence (OR = 1.05, 95% CI: 1.04–1.07) and carotid plaque number (one- to two-plaque group: OR = 1.04, 95% CI: 1.02–1.06; three-or-more-plaque group: OR = 1.09, 95% CI: 1.07–1.12). When baPWV was classified into quartiles, with the lowest quartile as reference, the ORs for having one, two, or three or more plaques increased in parallel with the quartiles of baPWV, indicating a dose-dependent effect. In a subgroup analysis, the association of baPWV and carotid plaque presence was more pronounced among younger participants (OR: 1.14 vs. 1.06 and 1.03 for the age groups <60 years, 60 ≤ 70 years, and ≥70 years, respectively, *P* for interaction <0.001). In a Chinese hypertensive population, baPWV was positively associated with carotid plaque presence and carotid plaque number. A more pronounced positive association between baPWV and carotid plaque presence was observed in younger participants.

## Introduction

Atherosclerotic cardiovascular disease (CVD) remains the most common cause of death worldwide [1]. In China, it is estimated that up to 290 million patients suffer from CVD [2]. Hypertension is the most important risk factor for CVD, though other risk factors contribute to the occurrence and development of the disease [3, 4]. Research has reported that the prevalence of CVD is much higher in the hypertensive population than in the general population [2]. Therefore, identifying asymptomatic subjects who are indeed at increased risk for CVD is extremely beneficial in hypertensive populations.

Carotid atherosclerosis, assessed by carotid plaque, provides direct evidence of systematic atherosclerosis. The presence of carotid plaque indicates the coexistence of atherosclerotic plaques elsewhere [5–7], and carotid plaque number can predict atherosclerotic plaque burden. Previous studies have revealed that the presence of carotid plaque and the number of carotid plaques are associated with increased risk of coronary artery disease and stroke [8–13]. In addition, their prediction value is stronger than that of other atherosclerotic markers, such as carotid intima–media thickness (CIMT) [13–15]. However, to capture a high-quality B-mode ultrasound image, well-trained operators are needed and therefore limit its use in undeveloped areas, such as rural China.

Arterial stiffness reflects degenerative changes of the extracellular matrix in the media layer and is characterized by elastin fatigue fracture and collagen deposition and cross-linking [16]. It is also one of the earliest detectable manifestations of adverse structural and functional changes within the vessel wall [17]. Elevated arterial stiffness, indicated by increased pulse wave velocity (PWV), has been suggested as an independent risk factor for cardiovascular mortality and morbidity [18–21]. Compared with carotid–femoral PWV (cfPWV), brachial–ankle PWV (baPWV) is an even simpler noninvasive and automatic measurement and provides a more convenient technique to evaluate artery stiffness, especially in large clinical trials. Previous studies have suggested that baPWV is significantly associated with the existence of atherosclerosis in the general male population, in end-stage renal disease subjects, in the middle-aged asymptomatic population and in the community-based population [22–25]. However, to our knowledge, no study has evaluated this association, specifically in the hypertensive population.

The current study aimed to investigate the relationship of arterial stiffness assessed by baPWV with carotid atherosclerosis, assessed by the presence of carotid plaque and carotid plaque number, in a Chinese hypertensive population. Moreover, since age and blood pressure are two dominant risk factors for both arterial stiffness and atherosclerosis [26–29], we evaluated the effect of age and level of blood pressure on the relationship between baPWV and carotid plaque.

## Methods

### Study population

All participants were from the China Stroke Primary Prevention Trial (CSPPT) population. Details about the CSPPT have been described in a previous publication [30]. Briefly, the CSPPT was a multicommunity, randomized, double-blind, controlled trial designed to evaluate the effect of folic acid therapy in reducing the risk of first stroke in Chinese hypertensive patients. The CSPPT showed that the combination of enalapril and folic acid is more effective in reducing the risk of first stroke than enalapril alone over a median follow-up period of 4.5 years.

**Fig. 1:**
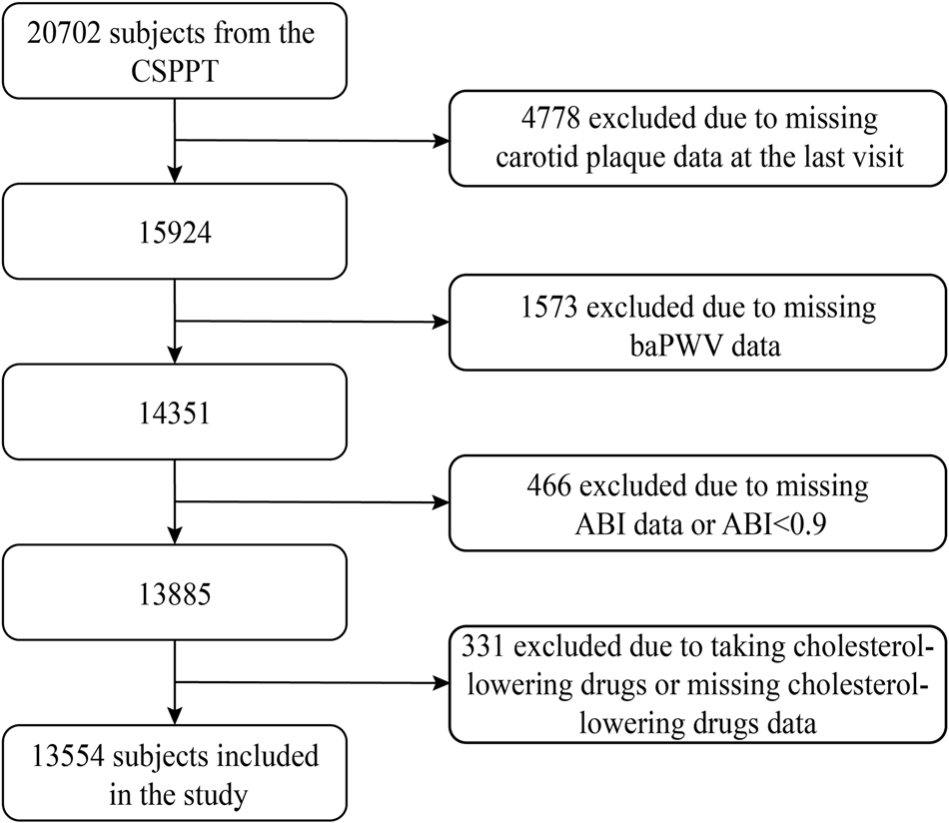
Flowchart of the study

Our study was a cross-sectional analysis of the CSPPT at the final visit. Overall, of the 20,702 subjects in the CSPPT, 14,351 subjects completed the final visit and had both carotid plaque data and baPWV data. Of those, 13,554 subjects without a background of peripheral artery disease (ankle brachial index ≥0.9) or cholesterol-lowering medication were included in the current study (see the flowchart in Fig. 1).

The parent study (CSPPT) and the current study were approved by the Ethics Committee of the Institute of Biomedicine, Anhui Medical University, Hefei, China. Written, informed consent was obtained from all participants before data collection.

### **Data** collection

#### Anthropometric data

Height, weight, waist circumference, and hip circumference were measured for each subject. Body mass index (BMI) was calculated as weight in kilograms divided by the square of height in meters. Blood pressure was measured with an electronic sphygmomanometer in a sitting position after resting for 5 min.

#### Questionnaire

All subjects were administered a standardized questionnaire. Information about demographics, occupation, medical history, medications, and personal habits such as smoking status and alcohol consumption was collected.

#### Laboratory tests

Overnight-fasted venous blood samples were collected from the participant’s forearm and samples were transported to the laboratory within 30 min. Blood samples were tested for fasting lipids (serum TC, HDL-c, and TG), fasting plasma glucose (FPG), plasma creatinine, and serum homocysteine using automatic clinical analyzers (Beckman Coulter) at the core laboratory of the National Clinical Research Center for Kidney Disease (Nanfang Hospital, Guangzhou, China). Serum folate was measured by a commercial laboratory using a chemiluminescent immunoassay (New Industrial). The estimated glomerular filtration rate (eGFR) was calculated using the Chronic Kidney Disease Epidemiology Collaboration equation [31].

#### Brachial–ankle pulse wave velocity

baPWV was measured by an oscillometry-based device (BP-203RPE III; Colin-Omron, Co., Ltd, Tokyo, Japan). Subjects were asked to remain in a supine position for 5 min with baPWV cuffs in place, after which baseline baPWV was measured and recorded by trained research staff. Two bilateral readings of baPWV measurements were simultaneously taken, and the maximum reading from each side was used for the analysis. More details describing the method of obtaining measurements have been published elsewhere [21].

#### Carotid ultrasonography

Carotid plaque was evaluated by certified sonographers using high-resolution B-mode ultrasonography (Terason 3000) with a 12L5A linear-array transducer. Carotid plaque was defined as a focal structure encroaching into the arterial lumen by at least 50% more than into the surrounding tissue, with a CIMT value of 0.5 mm or >1.5 mm thickness from the intima–lumen interface to the media–adventitia interface at the level of the internal carotid artery, common carotid artery, and bifurcation [32]. Carotid plaque number was defined as the sum of the total plaques on the left and right carotid arteries.

#### Covariables

The covariables included in all analyses were age, sex, BMI, systolic blood pressure (SBP), and diastolic blood pressure (DBP), total cholesterol (TC), high-density lipoprotein cholesterol (HDL-c), triglycerides (TG), FPG, eGFR, folate, homocysteine, antihypertensive medications, glucose-lowering medications, smoking status, alcohol consumption, folic acid treatment group, and study center.

#### Statistical analysis

Analyses were performed by EmpowerStats (http://www.empowerstats.com) and the statistical package R (3.2.3 version). Continuous variables are presented as means with standard deviations (SD), while categorical variables are presented as numbers and percentages. Characteristics of subjects were stratified by sex. Simple comparisons were performed between groups using Student’s *t* tests for continuous variables and the *χ*^2^ test for categorical variables. baPWV was analyzed as a continuous variable and divided into quartiles, with the lowest quartile as the reference class. Carotid plaque number was analyzed as a categorical variable consisting of four groupings: no-plaque group, one-plaque group, two-plaque group, and three-or-more-plaque group. Multivariate logistic regression was used to assess the relationship between baPWV and carotid plaque presence. Multinomial logistic regression was used to analyze the relationship between baPWV and carotid plaque number. Trend tests were calculated by transforming baPWV quartiles into a continuous variable. In the stratified analysis, possible modifications of the association between baPWV and carotid plaque presence were assessed for variables including age (<60 vs. 60 ≤ 70 vs. ≥70 years), sex, BMI (<24 kg/m^2^ vs. ≥24 kg/m^2^), smoking (never vs. former vs. current), alcohol drinking (never, former, current), treatment group (enalapril group vs. enalapril and folic acid group), SBP (<140 vs. ≥140 mmHg), TCHO (<5.2 mmol/l vs. ≥5.2 mmol/l), homocysteine (<15 µmol/l vs. ≥15 µmol/l), and study center (Anqing vs. Lianyungang). Multivariate logistic regression models were used in the analysis of interaction. A two-sided *P* value < 0.05 was considered significant.

## Results

### Characteristics of the study participants

The present study consisted of 13,554 hypertensive subjects with an average (SD) age of 64.3 (7.4) years. The demographic and clinical characteristics of the study subjects stratified by sex are listed in Table 1. The mean (SD) ages of male and female participants were 65.5 (7.4) years and 63.6 (7.3) years, respectively. Male subjects, totaling 5478 (40.4%), were significantly older, were more likely to be current smokers and current alcohol drinkers, had higher levels of DBP and serum homocysteine, and had a higher percentage of plaque prevalence but lower BMI, baPWV, SBP, eGFR, TC, TG, and folate levels compared with females. No significant differences were found in HDL-c and FPG levels.

**Table 1:** Characteristics of study subjects^a^

	Total (*n* = 13,554)	Gender		*P* value
		Male (*n* = 5478)	Female (*n* = 8076)	
Age, year	64.3 (7.4)	65.5 (7.4)	63.6 (7.3)	<0.001
Body mass index, kg/m^2^	24.9 (3.8)	24.1 (3.5)	25.5 (3.8)	<0.001
baPWV, m/s	17.2 (3.4)	17.2 (3.4)	17.3 (3.4)	0.006
Plaque presence, *N* (%)				<0.001
No	7893 (58.2)	2692 (49.1)	5201 (64.4)	
Yes	5661 (41.8)	2786 (50.9)	2875 (35.6)	
Plaque number, *N* (%)				<0.001
1	2575 (19.0)	1112 (20.3)	1463 (18.1)	
2	1467 (10.8)	702 (12.8)	765 (9.5)	
≥3	1619 (11.9)	972 (17.7)	647 (8.0)	
SBP, mmHg	135.4 (17.4)	132.8 (16.9)	137.2 (17.5)	<0.001
DBP, mmHg	82.0 (10.9)	82.3 (11.3)	81.8 (10.6)	0.007
Current smoking, *N* (%)	2892 (21.4)	2690 (49.2)	202 (2.5)	<0.001
Current drinking, *N* (%)	3032 (23.2)	2667 (52.7)	365 (4.6)	<0.001
Laboratory results, mmol/l				
Total cholesterol	5.3 (1.1)	5.1 (1.0)	5.5 (1.1)	<0.001
Triglycerides	1.8 (1.4)	1.5 (1.4)	1.9 (1.5)	<0.001
HDL cholesterol	1.3 (0.3)	1.3 (0.3)	1.3 (0.3)	0.096
Fasting glucose	6.2 (2.0)	6.2 (2.0)	6.3 (2.0)	0.398
Folate, ng/ml	18.9 (15.4)	18.4 (16.0)	19.3 (15.0)	<0.001
Serum homocysteine, μmol/l	13.5 (7.2)	15.3 (9.1)	12.3 (5.2)	<0.001
eGFR, mL/(min·1.73 m^2^)	88.6 (14.9)	87.1 (15.3)	89.7 (14.5)	<0.001
Medication use, *N* (%)				
Antihypertensive drugs	13,148 (97.1)	5327 (97.3)	7821 (97.0)	0.276
Glucose-lowering drugs	727 (5.5)	209 (3.9)	518 (6.6)	<0.001

*BMI* body mass index, *DBP* diastolic blood pressure, *eGFR* estimated glomerular filtration rate, *SBP* systolic blood pressure

^a^Variables are presented as mean (SD) or *n* (%)

### Association of baPWV and carotid plaque presence

Table 2 shows the results from the multivariate logistic regression analyses between baPWV and carotid plaque presence. Continuous baPWV was positively correlated with carotid plaque presence (odds ratio (OR) = 1.05, 95% CI: 1.04–1.07, *P* < 0.001) after adjusting for confounding factors, including age, sex, BMI, SBP, DBP, alcohol consumption status, smoking status, TCHO, TG, HDL-c, FPG, eGFR, homocysteine, folate, antihypertensive medication, glucose-lowering medication, study center, and folic acid treatment group. When baPWV was classified into quartiles, compared with the lowest quartile (Q1: <14.81 m/s), quartile 2 (Q2: 14.81 ≤ 16.73 m/s), quartile 3 (Q3: 16.73 ≤ 19.06 m/s), and quartile 4 (Q4: ≥19.06 m/s) were all positively correlated with carotid plaque presence (*P* < 0.001 for trend). The OR of carotid plaque presence increased in parallel with the quartiles of baPWV (ORs (95% CI): 1.06 (0.95, 1.19), 1.32 (1.17, 1.49), and 1.49 (1.31, 1.70) from the second to the fourth quartiles, respectively) after adjusting for the confounding factors mentioned above.

**Table 2:** Multiple analysis of baPWV and plaque presence

baPWV, m/s	*N*	Plaque presence, *N* (%)	Nonadjusted		Adjusted	
			OR (95% CI)	*P* value	OR (95% CI)	*P* value
Continuous	13,554	5661 (41.8)	1.12 (1.10, 1.13)	<0.001	1.05 (1.04, 1.07)	<0.001
Quartile						
Q1 (<14.81)	3389	1044 (30.8)	Ref.		Ref.	
Q2 (14.81 ≤ 16.73)	3378	1240 (36.7)	1.30 (1.18, 1.44)	<0.001	1.06 (0.95, 1.19)	0.317
Q3 (16.73 ≤ 19.06)	3384	1547 (45.7)	1.89 (1.71, 2.09)	<0.001	1.32 (1.17, 1.49)	<0.001
Q4 (≥19.06)	3403	1830 (53.8)	2.61 (2.37, 2.89)	<0.001	1.49 (1.31, 1.70)	<0.001
*P* for trend				<0.001		<0.001

Adjusted for age, gender, BMI, alcohol consumption status, smoking status, SBP and DBP at baseline, TC, HDL-c; FPG, eGFR, homocysteine, folate, TG, glucose-lowering medication, antihypertensive medication, study center, and folic treatment group

*BMI* body mass index, *SBP* systolic blood pressure, *DBP* diastolic blood pressure, *TC* total cholesterol, *HDL-c* high-density lipoprotein cholesterol, *FPG* fasting plasma glucose, *eGFR* estimated glomerular filtration rate, *TG* triglyceride

### Association of baPWV and carotid plaque number

Table 3 shows the results from the multinomial logistic regression analyses between baPWV and carotid plaque number. Continuous baPWV was positively correlated with carotid plaque number in each plaque number grouping after adjusting for confounding factors. Compared with the OR of the no-plaque group, the ORs (95% CIs) of having one plaque, two plaques, and three or more plaques were 1.04 (1.02, 1.06), 1.04 (1.02, 1.06), and 1.09 (1.07, 1.12), respectively. When baPWV was classified into quartiles with Q1 in each plaque group as a reference, Q2, Q3, and Q4 were all positively correlated with carotid plaque number (*P* < 0.05 for trend). In the one-plaque group, the ORs (95% CIs) were 1.03 (0.89, 1.18), 1.15 (0.99, 1.33), and 1.27 (1.08, 1.50) from Q2 to Q4, respectively. In the two-plaque group, the ORs (95% CIs) were 1.07 (0.88, 1.30), 1.47 (1.22, 1.79), and 1.45 (1.17, 1.79) from Q2 to Q4, respectively. In the three-or-more-plaques group, the ORs were 1.19 (0.96, 1.46), 1.67 (1.36, 2.06), and 2.20 (1.77, 2.74) from Q2 to Q4, respectively. In addition, in each quartile, compared with the no-plaque group, the ORs increased as the plaque number increased.

**Table 3:** Multinomial analysis of baPWV and carotid plaque number

baPWV, m/s	One plaque		Two plaques		Three or more plaques	
	aOR (95% CI)	*P* value	aOR (95% CI)	*P* value	aOR (95% CI)	*P* value
Continuous	1.04 (1.02, 1.06)	<0.001	1.04 (1.02, 1.06)	<0.001	1.09 (1.07, 1.12)	<0.001
Quartiles						
Q1 (<14.81)	Ref.		Ref.		Ref.	
Q2 (14.81 ≤ 16.73)	1.03 (0.89, 1.18)	0.708	1.07 (0.88, 1.30)	0.496	1.19 (0.96, 1.46)	0.111
Q3 (16.73 ≤ 19.06)	1.15 (0.99, 1.33)	0.072	1.47 (1.22, 1.79)	<0.001	1.67 (1.36, 2.06)	<0.001
Q4 (≥19.06)	1.27 (1.08, 1.50)	0.005	1.45 (1.17, 1.79)	<0.001	2.20 (1.77, 2.74)	<0.001
*P* for trend		0.002		<0.001		<0.001

Adjusted for age, gender, BMI, alcohol consumption status, smoking status, SBP and DBP at baseline, TC, HDL-c; FPG, eGFR, homocysteine, folate, TG, glucose-lowering medication, antihypertensive medication, study center, and folic treatment group

*aOR* adjusted odds ratio, *BMI* body mass index, *SBP* systolic blood pressure, *DBP* diastolic blood pressure, *TC* total cholesterol, *HDL-c* high-density lipoprotein cholesterol, *FPG* fasting plasma glucose, *eGFR* estimated glomerular filtration rate, *TG* triglyceride

### Assessment of interaction

**Fig. 2:**
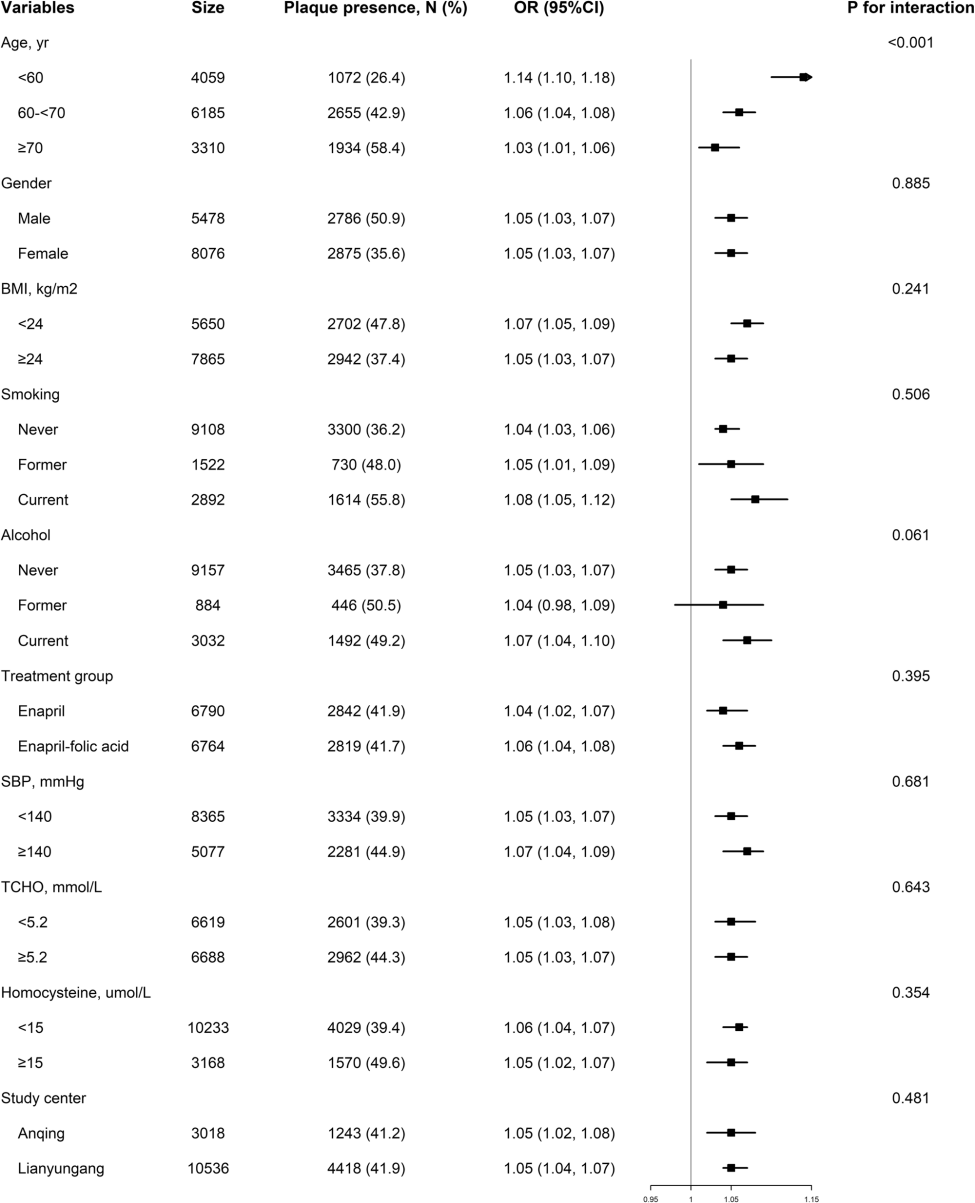
Forest plots of the association between baPWV and plaque presence in various subgroups. Adjusted, if not stratified, for age, sex, body mass index (BMI), alcohol consumption status, smoking status, systolic, and diastolic blood pressure (SBP and DBP) at baseline, total cholesterol (TC), high-density lipoprotein cholesterol (HDL-c); fasting plasma glucose (FPG), estimated glomerular filtration rate (eGFR), homocysteine, folate, triglycerides (TG), glucose-lowering medication, antihypertensive medication, study center, folic treatment group

Figure 2 shows the results of modification effects between baPWV and carotid plaque presence in different subgroups. The association of baPWV and carotid plaque presence was more pronounced among younger participants (OR: 1.14 vs. 1.06 and 1.03 for the age groups <60 years, 60 ≤ 70 years, and ≥70 years, respectively, *P* for interaction <0.001). To verify the effect of age, we explored the effect of sex on the age-related difference of this association and found that in both males and females, the association was stable (see Supplementary Fig. 1). There was no significant modification effect in any other subgroup, including sex, BMI, SBP, alcohol consumption status, smoking status, TC, homocysteine, study center, and folic acid treatment group (all *P* for interaction >0.05).

## Discussion

In this large-scale, population-based, cross-sectional study, we investigated the relationship of baPWV with carotid plaque presence and carotid plaque number in a Chinese hypertensive population. We demonstrated that baPWV was positively associated with carotid plaque presence and carotid plaque number. This association was independent of age, blood pressure, and other conventional cardiovascular risk factors. Moreover, we have shown for the first time that the association of baPWV and carotid plaque presence is more robust in relatively younger subjects.

Several published papers have investigated the association of arterial stiffness assessed by baPWV and carotid atherosclerosis. Kubozono et al. [22] found that high baPWV had a strong impact on early carotid atherosclerosis (defined as CIMT ≥1.0 mm) in a Japanese general male population. Munakata et al. [23] reported that higher baPWV was associated with greater max CIMT or carotid plaque score in patients with end-stage renal disease. Joo et al. [24] revealed that in a middle-aged asymptomatic population, subjects with higher baPWV had a higher prevalence of carotid plaque. Yang et al. [25] demonstrated that baseline baPWV is independently associated with the risk of new carotid plaque formation in a community population.

The studies above have confirmed that baPWV is an independent risk factor for atherosclerosis in the general male population, in end-stage renal disease subjects, in the middle-aged asymptomatic population, and in the community-based population. Our study adds new evidence for an association in a hypertensive population. The sample size in published studies is relatively small, which might have led to little power to disclose the associations. In addition, Kubozono et al. and Munakata et al. used CIMT instead of carotid plaque to reflect atherosclerosis, while previous studies have shown that carotid plaque is superior to CIMT in predicting cardiovascular events.

In our study, we found a positive association between baPWV and carotid plaque presence, and there was a dose-dependent effect. In the highest quartile of baPWV, the risk of carotid plaque presence was 1.49-fold higher than in subjects in the first quartile even after adjusting for confounding factors. When considering the amplitude of this association, an average increase in baPWV of 1 m/s corresponds to a 5% increased risk of carotid plaque presence. These findings are consistent with Joo et al. [24]. However, Joo et al. found that a 1 m/s increase in baPWV corresponded to a 38% increase in carotid plaque presence, which is much higher than the data in our study (5%). A reason for this difference might be that participants in Joo et al. were younger and asymptomatic. Fewer confounding factors may bring out a stronger association between baPWV and carotid plaque presence. We also found that baPWV was positively associated with carotid plaque number. Compared with the lowest quartile of baPWV, ORs in the highest quartile were 1.27 (1.08, 1.50), 1.45 (1.17, 1.79), and 2.20 (1.77, 2.74) in the one-plaque group, the two-plaque group, and the three-or-more-plaque group, respectively. That is, the risk of having more plaques increases with a higher baPWV.

In addition, we disclosed that the association between baPWV and carotid plaque presence was stronger in younger participants than in older participants (*P* for interaction <0.001). In the <60-year-old group, a 1 m/s increase in baPWV corresponded to a 14% increase in carotid plaque presence. In the ≥70-year group, however, the strength decreased to 3%. There are a few possible explanations for the age differences in this association. First, compared with younger subjects, older subjects had a higher prevalence of diabetes mellitus, hypercholesteremia, and other cardiovascular risk factors in this study (data not shown). BaPWV is only one of the determining factors for carotid plaque. More existing confounders may have weakened the association between baPWV and carotid plaque presence in the older population. Second, in the present study, the mean baPWV was lower in younger subjects (~15.6 m/s vs. 17.3 m/s vs. 19.2 m/s in age groups <60 years, 60 ≤ 70 years, and ≥70 years, respectively). A 1 m/s increase in baPWV would lead to a 6.4% change in the age group <60 years, a 5.8% change in the age group 60 ≤ 70 years, and a 5.2% change in the age group ≥70 years. Relatively larger changes in baPWV in younger subjects might lead to larger changes in carotid plaque presence.

Besides age, we tested for modification effects in different subgroups, including sex, SBP, BMI, etc. However, no additional interactions were found. Initially, we hypothesized that blood pressure might act as a modifier in the association between baPWV and carotid plaque presence because it has been reported that age and blood pressure are two dominant risk factors for both arterial stiffness and atherosclerosis [26–29]. However, the blood pressure subgroup did not show a modification effect (*P* for interaction is 0.681). One possible reason might be that the survey was conducted at the last visit of CSPPT. Therefore, 97.1% of the study subjects accepted antihypertensive medication, and for two-thirds of the subjects, blood pressure measurements were within the normal range (<140/90 mmHg). As a result, the underlying modification effect might be disguised.

The findings in the current study are of great clinical significance. Carotid plaque has more clinical predictive value for CVD than baPWV. However, in rural areas in China, the gap between skilled ultrasonic operators is large. Unlike ultrasonography, measurement of baPWV could be carried out by untrained staff. It could be used as a screening tool for obtaining a rough overview of carotid atherosclerosis and identifying hypertensive subjects with a high risk of CVD.

As for the underlying mechanisms explaining the positive association of baPWV and carotid plaque, it has been proven that arterial stiffness could be related to atherosclerosis through endothelial dysfunction, mechanical force on the inner wall of blood vessels, extracellular matrix disorder, elevated endothelial permeability, and vascular aging [16, 33]. Meanwhile, the presence of atherosclerosis also decreases the compliance of blood vessels, leading to stiffening of the arteries [34]. Arterial stiffness and atherosclerosis often coexist in the same vascular territories and share similar risk factors. Therefore, arterial stiffness and atherosclerosis may interact with each other. Atherosclerosis may not only be a consequence of arterial stiffening but may also increase arteriosclerosis in its advanced stage [16]. More exact explanations of their association remain to be explored.

In this study, baPWV was used for arterial stiffness assessment. cfPWV is also a well-accepted method in arterial stiffness measurement; baPWV is the most used in Asia and cfPWV in European and American studies [35–37]. Lu et al. [38] indicated that cfPWV was superior to baPWV in association with asymptomatic hypertensive target organ damage in the community-dwelling elderly Chinese population. Despite some differences in predictive value, both cfPWV and baPWV are strongly linked with cardiovascular disease and all-cause mortality, and are were highly correlated [19, 39–42]. In addition, the convenient measuring process and better reproductivity make baPWV more popular in large-scale investigations.

In conclusion, we found that in a Chinese hypertensive population, baPWV was positively associated with the presence of carotid artery plaque and plaque number. A more pronounced positive association between baPWV and the prevalence of carotid artery plaque was observed in younger participants.

## Limitations

Several limitations of our study should be noted. First, as a cross-sectional study, this study cannot clarify a causal relationship between baPWV and carotid plaque. Second, arterial stiffness was measured by baPWV in our study, and whether the association of cfPWV and carotid plaque is similar to our findings needs to be further explored. Third, our study is a part of the CSPPT, a randomized, controlled trial comparing the effect of enalapril and enalapril-folic acid on the primary prevention of stroke. Therefore, the proportion of subjects receiving antihypertensive medications and the rate of achieving optimal blood pressure in our study will be much higher than in the real world.

## Supplementary information

Supplementary Figure legends

Supplementary Figure1
