# Supplementary material for: Association of brachial–ankle pulse wave velocity and carotid plaque in Chinese hypertensive adults: effect modification by age
Source: Hypertens Res. 2020 Apr 17;43(8):808–16. doi: 10.1038/s41440-020-0432-2 (PMC7363666; doi:10.1038/s41440-020-0432-2)
Supplement: Supplementary file 1 — Supplementary Figure legends [file 41440_2020_432_MOESM1_ESM.docx]

Supplemental figure 1. Forest plots of the effect of sex on the age interaction of the association between baPWV and plaque presence. Adjusted for body mass index (BMI), alcohol consumption status, smoking status, systolic and diastolic blood pressure (SBP and DBP) at baseline, total cholesterol (TC), [high-density lipoprotein](file:///D:/enwiki/High-density_lipoprotein)-cholesterol (HDL-c); fasting plasma glucose (FPG), estimated glomerular filtration rate (eGFR), homocysteine, folate, triglycerides (TG), glucose-lowering medication, anti-hypertensive medication, study center, folic treatment group.
