# Supplementary figures and images for: Association of brachial–ankle pulse wave velocity and carotid plaque in Chinese hypertensive adults: effect modification by age
Source: Hypertens Res. 2020 Apr 17;43(8):808–16. doi: 10.1038/s41440-020-0432-2 (PMC7363666; doi:10.1038/s41440-020-0432-2)

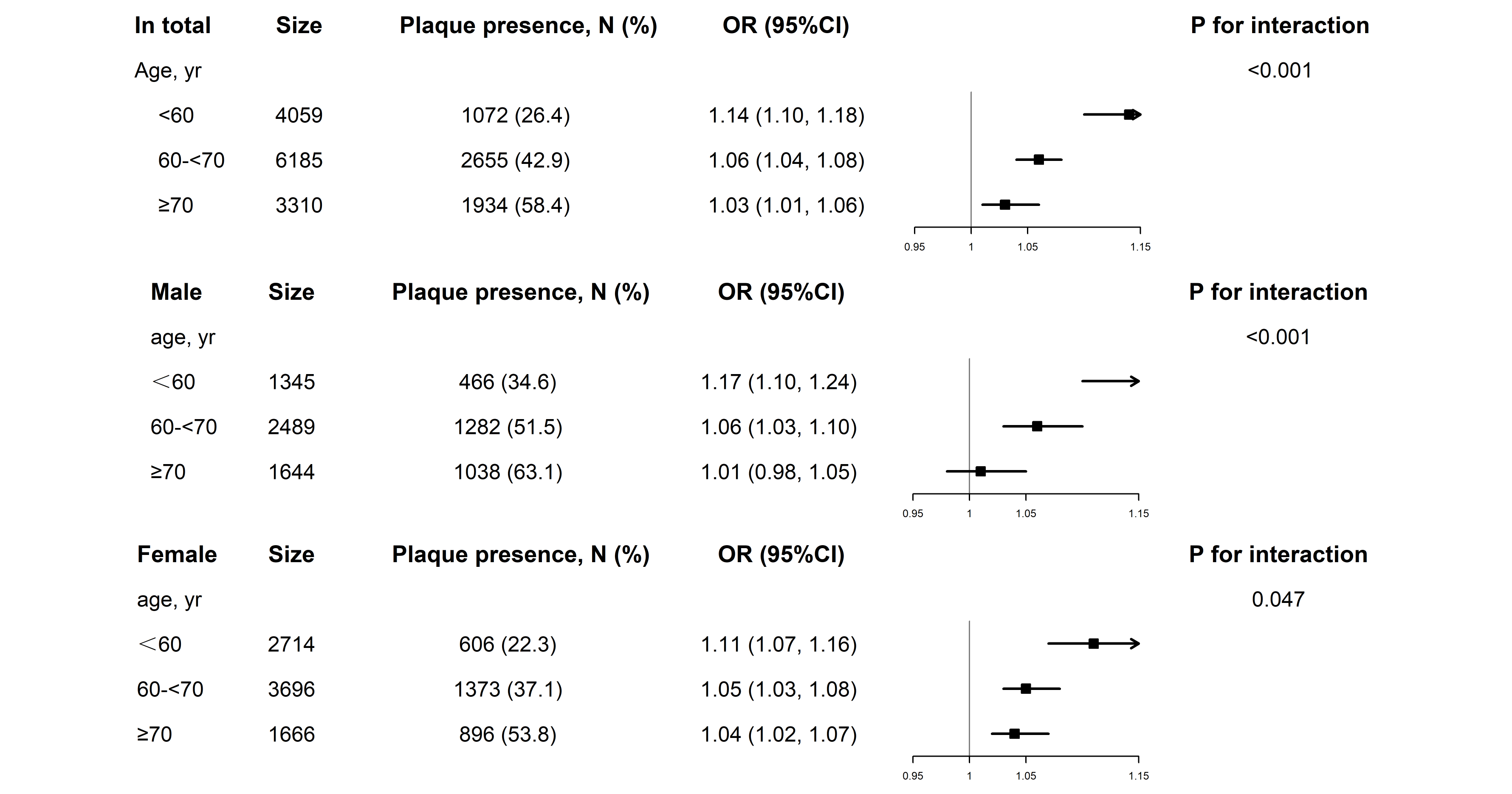

Supplement: Supplementary file 2 — Supplementary Figure1 [file 41440_2020_432_MOESM2_ESM.tif]
